# Population Structure of the Jewel Scarab *Chrysina gloriosa*

**DOI:** 10.1093/gbe/evag123

**Published:** 2026-05-20

**Authors:** Terrence Sylvester, Carl E Hjelmen, Michelle J Thompson, Leslie T Blackmon, James M Alfieri, Zachary Hoover, Tahmineh Esfandani, Heath Blackmon

**Affiliations:** Department of Biology, Texas A&M University, College Station, TX 77843, USA; Department of Biology, University of Memphis, Memphis, TN 38111, USA; Department of Biology, Texas A&M University, College Station, TX 77843, USA; Department of Biology, Utah Valley University, Orem, UT 84058, USA; Department of Biology, Texas A&M University, College Station, TX 77843, USA; Department of Biology, Texas A&M University, College Station, TX 77843, USA; Department of Biology, Texas A&M University, College Station, TX 77843, USA; Interdisciplinary Program in Ecology and Evolutionary Biology, Texas A&M University, College Station, TX 77843, USA; Department of Biology, Texas A&M University, College Station, TX 77843, USA; Department of Biochemistry and Biophysics, Texas A&M University, College Station, TX 77843, USA; Department of Biology, Texas A&M University, College Station, TX 77843, USA; Department of Biology, Texas A&M University, College Station, TX 77843, USA; Interdisciplinary Program in Ecology and Evolutionary Biology, Texas A&M University, College Station, TX 77843, USA

**Keywords:** sky islands, *Chrysina*, gene flow, Coleoptera, demography

## Abstract

The sky-island mountain ranges of the southwestern United States offer a natural setting for examining how climate and geography impact population structure and gene flow. The jewel scarab, *Chrysina gloriosa*, is a charismatic beetle restricted to high-elevation habitats in these mountains, where isolation and environmental change may be driving population divergence. We used low-coverage whole-genome resequencing and species distribution modelling to study population structure, gene flow, and demographic history across five mountain ranges in Arizona and West Texas. Our results indicate strong genetic differentiation among most populations, with recent gene flow detected only between two neighboring ranges, possibly through male-mediated dispersal. Demographic analyses reveal a decline in effective population size following late Pleistocene climate shifts, consistent with habitat contraction inferred from paleo-vegetation records. Future climate projections suggest further habitat loss and increasing isolation. Together, these results show how past and ongoing climatic changes have and continue to shape population structure in *C. gloriosa*, with important implications for its long-term evolutionary potential. These findings mirror patterns documented across sky-island taxa globally, supporting a general model in which climate-driven habitat dynamics and dispersal limitation interact to generate recurrent cycles of population fragmentation, genetic erosion, and demographic instability in montane systems.

Significance The sky-island mountain ranges provide a powerful context for testing how climate history and geographic isolation shape genome evolution. Using whole-genome resequencing of the jewel scarab *Chrysina gloriosa*, we show that populations are strongly differentiated among ranges, with evidence of limited recent gene flow and long-term demographic contraction following late Pleistocene climate shifts. These findings highlight the evolutionary consequences of isolation in fragmented habitats and demonstrate how integrating genomics with environmental history can illuminate the processes driving population divergence and persistence.

## Introduction

The sky-island mountain ranges of the southwestern United States and northern Mexico provide a classic system for studying how climate, geography, and dispersal shape population divergence (Knowles 2001; Riddle and Hafner 2006; McCormack and Smith 2008). These isolated mountain ranges harbor many species with fragmented distributions and limited gene flow, creating strong opportunities for local adaptation and evolutionary divergence (Van Devender et al. 1985; Wiens et al. 2019). Climatic oscillations during the late Pleistocene and Holocene have further shaped the history of these populations by driving the expansion and contraction of suitable habitats.

The jewel scarab *Chrysina gloriosa* (Cazier 1951; Young 1957; Moore et al. 2017) is one of four *Chrysina* species whose range extends into the United States, with populations found in West Texas, New Mexico, and southeastern Arizona. Adults are striking in appearance, with metallic green elytra marked by silver stripes, and are highly sought after by collectors (Genoways and Baker 1979). *Chrysina gloriosa* is closely associated with juniper woodlands, where adults feed and mate on juniper trees (Ritcher 1966; Morón 1990). Larvae develop in decaying wood, further linking the species to woodland habitats. Unlike the other three US *Chrysina* species, which typically occupy higher elevations and are associated with oak, walnut, and pine (Morón 1990), *C. gloriosa* occurs at lower elevations, down to 1,000 m, with a distribution strongly tied to juniper availability. The species’ current distribution is fragmented across isolated mountain ranges, consistent with the broader biogeographic pattern seen in many sky-island taxa (Van Devender et al. 1985). Historical evidence from packrat middens indicates that juniper was once more broadly distributed across the region during cooler and wetter Pleistocene climates (King and Van Devender 1977), suggesting that *C. gloriosa* may have experienced a more continuous range in the past. In addition to climate-driven habitat changes, *C. gloriosa* faces potential pressures from over-collection, with heavy collection pressure noted since the mid-20th century (Young 1957; Genoways and Baker 1979). Despite this ecological and evolutionary interest, little is known about the population structure, gene flow, or demographic history of *C. gloriosa*. The recent availability of a chromosome-level genome assembly for the species (Sylvester et al. 2024) now provides an opportunity to address these questions using modern genomic approaches.

Given the fragmented distribution of suitable habitat across the sky-island region, we tested alternative hypotheses regarding the drivers of population structure in *C. gloriosa*. We hypothesized that the mountain ranges act as primary barriers to dispersal, predicting strong genetic differentiation among ranges and patterns consistent with isolation-by-distance. Alternatively, we also hypothesized that within-mountain connectivity is primarily determined by the distribution of juniper woodland habitat, predicting greater gene flow where suitable habitat is continuous and genetic structure shaped by isolation-by-environment. Using genome-wide resequencing and species distribution modelling, we evaluate the relative influence of geographic distance and habitat continuity on gene flow within and between mountain ranges.

In this study, we use low-coverage whole-genome sequencing and species distribution modelling to address three key questions: (1) How structured are current *C. gloriosa* populations across the sky islands of Arizona and West Texas? (2) Is there evidence of ongoing gene flow between these populations? (3) How have historical climate changes shaped population size and connectivity?

By integrating genomic data with climate-based species distribution models, we provide new insights into how climate and geography shape the evolutionary dynamics of an iconic sky-island species.

## Results

### Sampling Summary

**Fig. 1.**
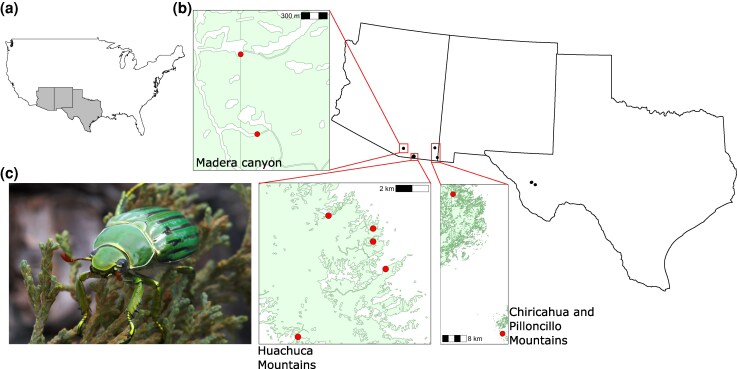
The sampling locations of *Chrysina gloriosa.* a) Broader geographic context of *C. gloriosa* distribution within the United States. The states where *C. gloriosa* occurs in the United States (Texas, New Mexico, and Arizona) are highlighted. b) The specimen sampling locations spanned two states in the United States (Texas and Arizona) and five mountain ranges. c) *C. gloriosa*, on a juniper branch. Credit: Alex Wild.

We collected a total of 81 *C. gloriosa* individuals across five mountain ranges in West Texas and Southeast Arizona during the summers of 2017, 2018, and 2019 (Fig. 1, Table S1). Our sampling was heavily sex biased, with 65 males and 16 females. Sample sizes varied across regions, with 7 individuals collected from the Chiricahua Mountains, 9 from the Peloncillo Mountains (Geronimo Trail), 13 from the Madera Canyon, and 11 from the Davis Mountains in West Texas. The highest number of individuals (41) was collected from the Huachuca Mountains, where we sampled four distinct sites: on the east side, Carr Canyon (16 individuals), Hunter Canyon (5), and Miller Canyon (10); and on the west side, Ida Canyon (10).

### Population Structure—Nuclear Genome

We used both non-model-based and model-based approaches to infer nuclear population structure in *C. gloriosa*, based on ∼380,000 nuclear single-nucleotide polymorphisms (SNPs) after filtering for linkage. Principal component analysis (PCA) revealed strong separation between Texas and Arizona populations along PC1 (22.3% variance explained), and within Arizona populations along PC2 (5.26%) (Fig. S1a). The *t*-distributed Stochastic Neighbor Embedding analysis (*t*-SNE), using the first five principal components, recovered more distinct clusters among Arizona populations, with clear separation between Chiricahua and Peloncillo samples, and a subdivision of Huachuca Mountain individuals into east and west groups (Fig. S1b).

**Fig. 2.**
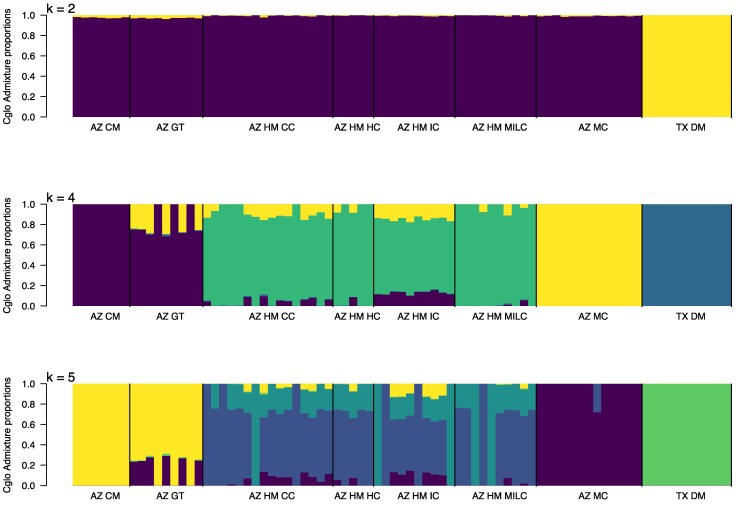
Bayesian clustering of *Chrysina gloriosa* populations with ADMIXTURE software using the nuclear variants. From top to bottom, bar plots of *K* = 2, 4, and 5.

Model-based inference using ADMIXTURE further supported this structure. At *K* = 2, samples are separated by state (Texas vs. Arizona) (Fig. 2). At *K* = 4, the Davis Mountains, Madera Canyon, Huachuca Mountains, and Chiricahua and Peloncillo Mountains emerged as distinct ancestral populations. However, admixture was evident in the Peloncillo and Huachuca populations. Within the Huachuca Mountains, Ida Canyon (west side) showed a distinct admixture pattern compared to the east side populations. These patterns remained consistent at *K* = 5.

To explore spatial connectivity, we estimated effective migration rates using Fast Estimation of Effective Migration Surfaces (FEEMS). High migration was inferred between the Chiricahua and Peloncillo Mountains, while reduced migration was detected between the east and west Huachuca Mountains and between Madera Canyon and the Huachuca range (Fig. S2).

### Population Structure—Mitochondrial Genome

To assess concordance between nuclear and mitochondrial variation, we repeated population structure analyses using mitochondrial SNPs. PCA recapitulated the strong Texas–Arizona split (PC1, 87.4%), but Arizona samples showed less separation along PC2 (Fig. S3a). The *t*-SNE analysis recovered finer resolution within Arizona samples. The Chiricahua and Peloncillo populations were grouped into their own clusters. The individuals from Huachuca and Madera Canyon were grouped into a single mixed cluster (Fig. S3b).

ADMIXTURE results diverged more strongly from nuclear patterns at higher *K* values (Fig. S4). At *K* = 4, the Davis Mountains, Madera Canyon, Huachuca Mountains, and Chiricahua + Peloncillo Mountains emerged as distinct ancestral populations. Peloncillo samples showed dominant ancestry from Chiricahua with some minor admixture from Madera Canyon populations. The Huachuca populations exhibited mixed ancestry from the ancestral populations of the Huachuca Mountains and Madera Canyon. At *K* = 5, Peloncillo emerged as a distinct ancestral population, with minor contributions from Madera Canyon and Chiricahua populations.

### Population Structure—F_ST_

Genetic differentiation among Arizona sky-island populations was uniformly low, with pairwise weighted *F*_ST_ values ranging from 0.006 to 0.015 (Table S2). This limited divergence indicates weak population structure within the Arizona region. In contrast, comparisons between Arizona and Texas populations revealed consistently high levels of differentiation, with weighted *F*_ST_ values ranging from 0.282 to 0.287 across all pairwise contrasts. Notably, the magnitude of divergence between Texas and each Arizona population was highly consistent, with minimal variation among comparisons. The global weighted *F*_ST_ across all populations was intermediate (0.131), reflecting the combined effects of weak within-Arizona structure and strong regional divergence. This pattern of uniformly low differentiation within Arizona and consistently high differentiation between Arizona and Texas populations indicates a hierarchical population structure. The lack of elevated *F*_ST_ among Arizona populations suggests recent fragmentation and/or ongoing connectivity among sky islands, whereas the pronounced and uniform divergence between regions supports a deeper, long-term separation of Texas populations from those in Arizona.

### Population Diversity

Nucleotide diversity (*π*) was highly similar across Arizona sky-island populations, with mean values ranging from 0.00048 to 0.00055 (Table S3). The Texas population exhibited a slightly higher value (*π* = 0.00063) but remained comparable in magnitude. No population showed evidence of reduced genetic diversity relative to others. The uniformity of *π* across Arizona populations indicates an absence of strong bottlenecks, inconsistent with expectations under recent founder events. Instead, the combination of comparable nucleotide diversity and low pairwise differentiation among Arizona populations supports a model of recent fragmentation of a historically connected population, with limited loss of genetic diversity.

### Demographic History

**Fig. 3.**
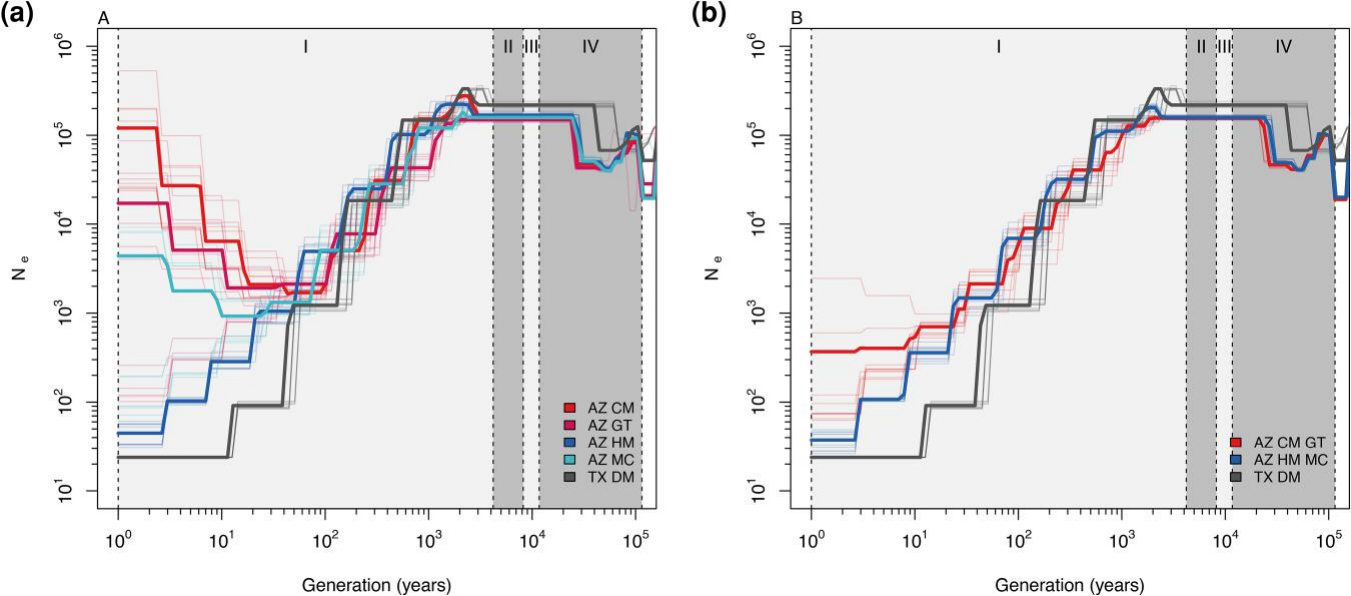
Demographic history of *Chrysina gloriosa*. AZ CM: Arizona Chiricahua Mountains, AZ GT: Arizona Geronimo Trail, AZ HM: Arizona Huachuca Mountains, AZ MC: Arizona Madera Canyon, and TX DM: Texas Davis Mountains. a) All populations are considered as independent populations. b) AZ CM and AZ GT populations are considered a single population, and AZ HM and AZ MC population are considered a single population. Gray shadings represent geological times: I: Late Holocene, II: Mid Holocene, III: Early Holocene, IV: Pleistocene.

To contextualize population structure in evolutionary time, we inferred demographic history using SMC++. All *C. gloriosa* populations exhibited a decline in effective population size from the late Pleistocene to mid-Holocene (Fig. 3a). Arizona populations show convergence in their demographic trajectories ∼10,000 years ago, consistent with a historically panmictic population that later fragmented. In contrast, the Texas population remained distinct throughout the time series, indicating long-term isolation.

When analyzed separately, some Arizona populations (Madera Canyon, Chiricahua, and Peloncillo) appeared to show recent increases in effective size. However, grouping geographically adjacent populations (e.g. Huachuca + Madera; Chiricahua + Geronimo Trail) removed these apparent expansions, suggesting they may be artifacts of recent gene flow rather than true demographic growth (Fig. 3b).

Maximum likelihood inference in momi2 provided a robust temporal framework for the divergence of *C. gloriosa* (Fig. S5). In our pairwise assessment within Arizona populations, we found that the divergence time between Chiricahua Mountain and Huachuca Mountain (245 years ago) and the divergence time between Geronimo Trail and Huachuca Mountain (385 years ago) were lower than when each of these populations was compared with the Madera Canyon population (665 and 601 years ago, respectively). Therefore, we tested three model scenarios where the population separation follows geography (M1: [Chiricahua + Geronimo Trail] + [Huachuca Mountain + Madera canyon]) and does not follow geography (M2: [[[Chiricahua + Geronimo Trail] + Huachuca Mountain] + Madera canyon]; M3: [[[Chiricahua + Geronimo Trail] + Madera canyon] + Huachuca Mountain]). Each model had four parameters. We tested the model fit by assessing the AIC, and M1 emerged as the best fit model (ΔAIC = AICM2—AICM1 3315.645; ΔAIC = AICM3—AICM1 3292.829).

Our analysis confirms that Chiricahua and Geronimo Trail populations function as a single panmictic unit, with estimated divergence times near zero, consistent with the absence of detectable structure in pairwise *F*_ST_ and the disappearance of apparent demographic expansions when these populations are pooled in SMC++. Within M1, Huachuca Mountain and Madera Canyon populations diverged approximately 486 years ago, and the [Chiricahua + Geronimo Trail] clade separated from [Huachuca + Madera Canyon] approximately 890 years ago. These divergence times are much older than our pairwise analysis, which is expected when joint demographic history is modelled simultaneously. The divergence of Arizona and Texas populations occurred approximately 56,000 years ago, consistent with Late Pleistocene climate cycles.

### Species Distribution Modelling

To assess how past and future climates may influence *C. gloriosa* distribution, we used MaxEnt to model habitat suitability across time periods (Last Interglacial–LIG, Last Glacial Maximum–LGM, Mid Holocene–MH, and current climate). Habitat suitability was divided into four bins based on the probability of occurrence: poor (60%< and ≤70%), fair (70%< and ≤80%), good (80%< and ≤90%), and excellent (90%<).

From LIG to the present, we observed a reduction in the total area of suitable habitat (by ∼5,300 km^2^), especially in poor, fair, and good suitability categories (Fig. S6). The area classified as excellent habitat (>90% occurrence probability) showed modest fluctuation across time periods, with a slight increase during the LGM, followed by contraction by the mid-Holocene.

Projections for 2021 to 2040 and 2061 to 2080 under modest and high greenhouse gas emission scenarios indicated further reductions in suitable habitat. While poor and fair habitats showed slight increases under some future scenarios, excellent habitats declined substantially, by ∼640 km^2^ under modest emissions and ∼900 km^2^ under high emissions (Fig. S7).

## Discussion

### Population Structure and Isolation Across the Sky Islands

Our analyses reveal strong genetic differentiation among *C. gloriosa* populations in the Sky Islands of Arizona and West Texas, with limited evidence of recent gene flow. Nuclear markers show that each mountain range generally harbors distinct genetic clusters, consistent with isolation by distance and landscape barriers. The only clear signal of recent gene flow is between the Chiricahua and Peloncillo Mountains, which are geographically proximate and separated by a relatively narrow valley. This pattern of isolation across mountain ranges aligns with findings in other sky-island taxa (Knowles 2001; Sekar and Karanth 2013; Tonzo and Ortego 2021; Hartley et al. 2022), where historical climate-driven range shifts and current habitat discontinuities have promoted population divergence. Given the strong habitat specialization of *C. gloriosa* on juniper woodlands, these barriers are likely to have an even stronger effect than in more generalist species.

### Convergent Patterns of Divergence Across Sky-Island Systems

Patterns of population structure in *C. gloriosa* closely mirror those documented across North American sky-island systems, while also highlighting taxon-specific differences in dispersal biology. Strong genetic subdivision among mountain ranges, coupled with limited gene flow across intervening lowlands, is consistent with Pleistocene-driven divergence observed in the flightless grasshopper (*Melanoplus oregonensis*), where high among-range differentiation and coalescent analyses supported multiple allopatric refugia (Knowles 2001). In contrast to the strict isolation in that system, we detect limited male-mediated gene flow between geographically proximate ranges, suggesting that even rare long-distance dispersal by volant insects can partially counteract isolation. At finer spatial scales, the subdivision of Huachuca Mountain populations parallels patterns observed in the Mexican jay (*Aphelocoma wollweberi*), where ecological gradients over short distances generate morphological and genetic differentiation despite ongoing gene flow (McCormack and Smith 2008). Together with broader phylogeographic work on the Mexican Plateau horned lizard (*Phrynosoma orbiculare*) (Bryson et al. 2012), these results underscore the joint influence of landscape history, topographic barriers, and dispersal capacity in structuring montane genetic diversity, including deep east–west divergence across the Chihuahuan Desert consistent with longstanding regional breaks (Riddle and Hafner 2006).

Demographic inference further indicates that *C. gloriosa* populations experienced postglacial contraction, concordant with paleo-ecological evidence for juniper retreat and with patterns widely reported across sky-island taxa (King and Van Devender 1977; Van Devender et al. 1985). The inferred fragmentation of a previously more continuous population aligns with models emphasizing distributional shifts, rather than strict vicariance, as primary drivers of divergence in montane systems (Knowles and Massatti 2017). These dynamics are consistent with the broader flickering connectivity framework, in which Pleistocene climatic oscillations alternately connect and isolate populations, although here with contraction during postglacial warming rather than glacial expansion ( [Bibr evag123-B14][Bibr evag123-B11]).

Comparisons across global sky-island systems reveal strong convergence in both demographic and evolutionary outcomes. Afroalpine plant lineages exhibit low within-population diversity and recent intermountain divergence despite substantial dispersal, paralleling the small effective population sizes and strong structure inferred here (Kandziora et al. 2022). Similarly, studies of European alpine floras show that repeated Plio-Pleistocene isolation can accelerate lineage accumulation while simultaneously increasing demographic vulnerability (Wootton et al. 2025). In *C. gloriosa*, however, the absence of evidence for recent diversification alongside pronounced population decline suggests a system in which fragmentation has progressed to the point of genetic erosion rather than lineage formation. Collectively, these results reinforce a general model in which climate-driven habitat dynamics, modulated by dispersal limitation and ecological specialization, generate recurrent patterns of strong differentiation and demographic instability across sky-island biota.

### Discordance Between Nuclear and Mitochondrial Markers

A notable finding is the discordance between nuclear and mitochondrial patterns of structure between the Chiricahua and Peloncillo populations. While nuclear data indicate recent gene flow, mitochondrial data do not. Given our male-biased sampling (81% males) and the flight behaviors of beetles, it is likely that this reflects male-mediated dispersal, as has been documented in other insect taxa (Lagisz et al. 2010). Male-biased gene flow across short distances could maintain some nuclear connectivity while mitochondrial lineages remain more structured. This pattern deserves further study, but it provides a useful comparison to vertebrate systems in the sky islands, where sex-biased dispersal also contributes to mito-nuclear discordance (Barth et al. 2013; Folt et al. 2019; Shu et al. 2022; Giannelli et al. 2026). The possibility of human-mediated movement, for example, via collectors, cannot be ruled out and warrants further investigation, although we consider natural dispersal the more likely explanation for the observed pattern.

### Demographic History Shaped by Climatic Change

The demographic history of *C. gloriosa* shows a marked decline in effective population size following the end of the last glacial period. This pattern is consistent across all Arizona populations and matches the known shift in regional vegetation documented in packrat midden studies (King and Van Devender 1977; Van Devender et al. 1985), where juniper woodlands contracted to higher elevations as temperatures rose. The demographic contraction we infer aligns with a broader pattern seen in sky-island species, where Pleistocene-Holocene climate transitions fragmented once-continuous populations (Knowles 2001; Rubidge et al. 2012; Knowles and Massatti 2017; Hartley et al. 2022). Interestingly, we find that the Texas and Arizona populations appear to have been isolated even before the last glacial maximum, suggesting long-term divergence between these regions, likely reflecting deep phylogeographic structure shaped by historical barriers to gene flow.

### Future Climate Impacts and Evolutionary Consequences

Our species distribution models predict a further reduction in suitable habitat for *C. gloriosa* under future climate scenarios. Projected increases in temperature and shifts in precipitation patterns are likely to drive additional habitat contraction and isolation. From an evolutionary perspective, these changes could accelerate genetic drift, increase inbreeding, and reduce adaptive potential in already small and fragmented populations. Similar concerns have been raised for other sky-island taxa (Zhang et al. 2019; Ceresa et al. 2024), where future climate change is expected to further erode connectivity and increase extinction risk. In *C. gloriosa*, which is highly dependent on juniper habitat and has limited dispersal ability, these effects may be especially pronounced.

### Broader Evolutionary Implications

These results contribute to a growing understanding of how climate history and geography shape divergence in sky-island systems. Like many vertebrates and some invertebrates studied in this region, *C. gloriosa* shows deep population structure, a strong signature of post-glacial demographic contraction, and evidence of restricted contemporary gene flow. The male-biased gene flow we observe between neighboring ranges adds a new dimension to our understanding of how insect populations may persist in fragmented landscapes, complementing other studies where sex-biased dispersal is often better documented (Lagisz et al. 2010). Moreover, the strong concordance between demographic inferences and paleoecological records underscores the value of integrating genomic and ecological data to reconstruct the evolutionary history of sky-island taxa. Insect populations remain underrepresented in this literature compared to birds, reptiles, and mammals, and studies such as this provide important comparative data for understanding how life history, dispersal capacity, and habitat specialization interact with climate-driven isolation across taxa.

### Conservation Considerations and Future Directions

While our primary focus is on evolutionary processes, these findings also have relevance for conservation. The effective population sizes we infer are low, and projected habitat losses raise concerns about the long-term persistence of isolated populations. Efforts to monitor populations, limit over-collection, and identify potential corridors for gene flow may help maintain genetic diversity. Future work should aim to expand sampling to additional mountain ranges, including those in Mexico, to better understand regional connectivity. Life history data, particularly on dispersal abilities and mating behavior, will also be critical for refining models of gene flow and divergence. Comparative genomic studies of other *Chrysina* species in the region could also provide valuable insight into how different ecological and behavioral traits influence patterns of divergence in this fascinating group.

## Conclusion

Our results allow us to evaluate the relative support for the two hypotheses framing this study. The strong genetic differentiation among mountain ranges, the pronounced Texas–Arizona divergence, and the pattern of isolation-by-distance within Arizona are broadly consistent with the topographic isolation hypothesis, in which mountain ranges act as primary barriers to dispersal. However, the signal of recent restricted gene flow within the Huachuca mountains (east and west populations) suggests that habitat connectivity also modulates gene flow at fine spatial scales. Together, these results suggest that topographic isolation is the dominant driver of population structure in *C. gloriosa*, but that ecological specialization on juniper habitat amplifies or attenuates connectivity in a spatially heterogeneous manner.

Our study shows that *C. gloriosa* populations in the sky islands exhibit strong genetic structure shaped by a combination of historical climate change and contemporary landscape barriers. Limited gene flow between ranges, sex-biased dispersal, and small effective population sizes point to an ongoing process of divergence that mirrors patterns seen in many vertebrate taxa in this system. As climate change continues to alter habitat availability, these populations may experience further isolation, with consequences for genetic diversity and evolutionary potential. More broadly, this work highlights the utility of integrative approaches, combining genomics, demographic modelling, and paleoecological context, for advancing our understanding of evolutionary dynamics in fragmented landscapes. Continued comparative studies across taxa and regions will be essential for developing a more general theory of climate-driven divergence in montane systems.

## Methods

### Sample Collection

We collected *C*. *gloriosa* samples from West Texas and Southeast Arizona during July and August of 2017, 2018, and 2019 (Fig. 1, Table S1). All specimens were collected at night between 7 PM and 11 PM. We used a combination of Mercury vapor and UV lamps placed in front of a vertical white sheet to attract beetles. All samples collected were stored in 100% ethanol at −20 °C until DNA extraction.

### DNA Extraction and Sequencing

We used muscle tissue dissected from the hind legs of the beetle to extract DNA using the QIAGEN Blood and Tissue DNA Extraction Kit (catalogue number: 69504, QIAGEN, Germantown, MD, USA), following the manufacturer's protocol. We used the NanoDrop (catalogue number: ND-ONE-W, Thermo Fisher, Waltham, MA) to assess the quality of the extracted DNA, and the Quantus fluorometer (catalogue number: E6150, Promega Corporation, Madison, WI) to quantify the extracted DNA. We sequenced DNA through the Texas A&M AgriLife Genomics and Bioinformatics service center (https://www.txgen.tamu.edu/) using the Illumina short-read sequencing platform and the NovaSeq 6000 sequencing system, generating 2 × 150 bp paired-end reads at 1 to 2× coverage.

### Mapping and Variant Calling

We combined the forward and reverse reads from each lane to generate a single forward-read file and a single reverse-read file for each specimen. Then we filtered adapter sequences and reads below a quality score of 20 using fastP (Chen et al. 2018). We assessed the quality of the reads pre- and post-filtering, using fastQC and combined all reports using multiQC for analysis (Simons 2010; Ewels et al. 2016). We discarded the unpaired reads and only kept paired reads for mapping and variant calling.

We indexed the *C. gloriosa* genome (containing both nuclear and mitochondrial genomes) using the faidx module of SAMtools version 1.14 and the index module of BWA version 0.7.17 (Li and Durbin 2009, 2010; Li 2013). We then mapped all reads against the genome using the BWA-MEM algorithm and converted the resulting SAM files to BAM format using the SAMtools view module. Next, we sorted the mapped files by name using the SAMtools sort module. Then, we used the SAMtools fixmate module to correct errors in read-pairing due to the alignment program, followed by the SAMtools markdup module to remove duplicate reads. Finally, we extracted mapped reads using the SAMtools view module. To remove spurious mappings, we only kept sites with a mapping quality greater than 30.

We used a combination of BCFtools version 1.14 and VCFtools version 0.1.16 for variant calling and filtering (Danecek et al. 2021, 2011). First, we generated the initial variant file using the BCFtools mpileup module. We called variants using the BCFtools call module with the multiallelic caller as the variant calling option. We kept single-nucleotide polymorphisms (SNPs) with a base quality of 20 and a mapping quality of 30. We separated the resulting VCF file into two subsets representing nuclear-only and mitochondrial-only variants. We further filtered the variants based on quality, read depth, missingness, and minor allele frequency. We used the following thresholds to filter variants: minor allele frequency (MAF) of 0.05 (5%), maximum missing percentage across all samples of 0.8 (tolerate 20% missing data), minimum read depth per SNP of 1x, and minimum read depth per SNP across all samples of 1x. We retained only bi-allelic single-nucleotide polymorphisms (SNPs) in our variant dataset. Our final SNP dataset comprised 2 million nuclear variants and 577 mitochondrial variants.

### Population Parameter Estimation

Population genetic differentiation among sky-island populations of *C. gloriosa* was quantified using the Weir and Cockerham estimator of *F*_ST_ as implemented in VCFtools v0.1.16 (Danecek et al. 2011). Analyses were conducted on filtered SNP dataset with consistent site and genotype filtering applied across all populations to minimize biases associated with missing data and allele frequency differences. Pairwise *F*_ST_ was calculated for all population combinations, and weighted estimates (*θ*) were used for interpretation, as these account for variation in sample size and allele frequencies across loci. In addition, a global *F*_ST_ was estimated across all populations to summarize overall genetic structure.

Nucleotide diversity (*π*) was estimated for each population using VCFtools v0.1.16 with a sliding window approach (10 kb windows), and genome-wide mean *π* was calculated by averaging windowed estimates to obtain per-population diversity measures.

### Population Structure and Demography of C. Gloriosa

We filtered the VCF file for population structure analysis to remove variants in linkage disequilibrium (LD) using PLINK version 1.9 (Chang et al. 2015). We used a sliding window of 50 SNPs, where we advanced by 10 SNPs. Additionally, we used a correlation coefficient (*r*^2^) of 0.1 to filter SNPs that are in LD. This process resulted in 380,605 SNPs in our filtered VCF file for the nuclear genome. We did not apply the LD filter for the mitochondrial genome since it is a non-recombining single genome, and all SNPs are linked.

We used several approaches to analyze population structure. First, we used the filtered SNPs to conduct a PCA. We used PLINK version 1.9 to perform the PCA and the R package tidyverse to visualize the results (Wickham et al. 2019). We then used *t*-SNE, a machine learning approach, on principal components from the PCA to further reduce dimensionality and explore fine-scale population structure. We set the perplexity parameter, which defines the number of neighbors for a given point, to 10 since we had 10 or fewer specimens from each location. We set the theta parameter to zero to increase the accuracy.

We also estimated the individual admixture proportions using ADMIXTURE version 1.3.0 in a maximum likelihood framework (Alexander et al. 2009). We used the filtered variant set to estimate individual admixture proportions. We set the *K*-value (ancestral populations) from 2 to 12 with a 10-fold cross-validation.

We used the program FEEMS version 1.0.0 to estimate the migration surface of *C. gloriosa* across Arizona (Marcus et al. 2021). FEEMS requires four input files: genotypes, locations, outer boundary, and a dense global grid. Based on our admixture results, we removed samples from Texas from our variant file, as there was no evidence for migration between samples from Texas and Arizona. This removal resulted in some variants becoming monomorphic. We further removed these variants from the variant file as the program FEEMS fails in the presence of monomorphic sites. For the outer boundary, we used the Arizona border coordinates.

We used PLINK version 1.9 for imputation of missing genotypes. For the missing sites, we assigned the mean genotype at each SNP. We used the R package dggridR to make a discrete triangular grid for Arizona (Barnes and Sahr 2017). Each triangle represents an area of approximately 25 km^2^. At this resolution, we can capture fine-scale migration patterns (e.g. those within mountains). We then ran cross-validation for a range of values for the smoothness parameter (*λ*), ranging from 0.1 to 10, and selected the *λ* with the least cross-validation error. The smoothness parameter determines the strength of the penalty placed on the migration surface, with lower values for *λ* revealing fine-scale migratory patterns (Marcus et al. 2021). Based on the cross-validation result, we chose 35.93 as the *λ* value and estimated the migratory surface with all other parameters kept at their default values.

We used SMC++ version 1.15.2 to estimate the demographic history for each population of *C. gloriosa* (Terhorst et al. 2017). First, we subset the multisample VCF file into population-level using the bcftools view module. Then we converted the VCF file to SMC format using the vcf2SMC module in SMC++. We created a separate SMC file for each individual and scaffold following the composite likelihood approach. The advantage of this approach is that we can incorporate information from all the individuals for a given location when we estimate the demographic history, instead of a single distinguished individual. We only used the SNPs from the largest 13 contigs, which capture 99.8% of the assembled genome. We then estimated the demographic history using all the SMC files for a given population using the estimate module in SMC++, and we repeated this process ten times for each population. We used a generation time of one year and a mutation rate of 2.1 × 10^−9^ to scale time and the effective population size. We used the mutation rate estimate from a recent publication on the non-biting midge, as we do not have any information on the genome-wide mutation rate for *C. gloriosa* (Oppold and Pfenninger 2017). The exact mutation rate estimate is used in a similar analysis of the Colorado potato beetle (Pélissié et al. 2022).

To complement the SMC++ trajectories and explicitly test the hypothesis that recent effective size increases in individual Arizona populations are artifacts of gene flow, we performed targeted demographic inference using momi2 version 2.1.15 (Kamm et al. 2020). We first assessed the split time of each Arizona population pair independently to find the initial population divergence pattern. We then constructed a full population divergence model, including the Texas population, to estimate maximum likelihood estimations of the convergence time of each population. We used the TNC (Truncated Newton) algorithm with randomized starting parameters as our likelihood estimation method. We considered alternative scenarios for within Arizona population fragmentation (ones that do not follow geography) and assessed the model fit using the Akaike information criterion. The tested population divergence scenarios were as follows: M1: ([Chiricahua + Geronimo Trail] + [Huachuca Mountain + Madera canyon]), M2: ([Chiricahua + Geronimo Trail] + [Huachuca Mountain] + [Madera canyon]), M3: ([Chiricahua + Geronimo Trail] + [Madera canyon] + (Huachuca Mountain)). Each model was run for 100 replicates with randomized starting parameters to assess model convergence.

### Species Distribution Modelling

We downloaded all available occurrence data for *C. gloriosa* from the GBIF dataset (accessed March 2021) (GBIF 2021). We removed data points that had no coordinates and duplicated data points. We supplemented this dataset with the Texas A&M insect collection datasets and our data from field visits, which yielded a final dataset consisting of 776 occurrences.

Next, we downloaded climatic data for paleo, current, and future climates from the Worldclim database (accessed May 2022). We used the Worldclim dataset version 2.0 to download current and future climatic data and version 1.4 to download paleoclimatic data (Fick and Hijmans 2017). All data had a resolution of 30 arc seconds except for the Last Glacial Maximum, which had a resolution of 2.5 arc minutes. Our paleoclimatic dataset included climatic data from the Last Interglacial (LIG), Last Glacial Maximum (LGM), and Mid-Holocene (MH) periods from the Community Climate System Model version 4 (CCSM4) (Gent et al. 2011). Our climatic data comes from two shared socio-economic pathways (SSPs). Namely, SSP245 and SSP585. These SSPs represent greenhouse gas emission scenarios and associated climatic conditions. SSP245 represents a modest emission scenario, and SSP585 represents the maximum emission scenario. We downloaded predicted climatic data for the period of 2021 to 2040 (near future) and 2061 to 2080 (far future). These future climatic data come from the climatic modelling at the Goddard Institute for Space Studies (GISS) (Kelley et al. 2020). Each data set for a given climatic condition consists of 19 bioclimatic variables. We also downloaded elevation data for the current climate through the WorldClim dataset.

We downloaded the individual shapefiles of Texas, New Mexico, and Arizona through the United States Census Bureau (https://www.census.gov/). We combined them to produce a single shapefile that uses ArcGIS to capture all three states. We then cropped the bioclimatic datasets to the extent of the study area and calculated the Pearson correlation between each bioclimatic variable using the R package raster (Hijmans et al. 2025). We randomly selected a single bioclimatic variable from those pairs with an absolute Pearson correlation coefficient greater than 0.7 for the species distribution modelling. This process retained only seven bioclimatic variables for modelling the species distribution.

We used MaxEnt version 3.4.4 to model the species distribution for the current, paleo (LIG, LGM, MH), and future climates (Phillips et al. 2004, 2006). We used the output format as Cloglog, which produces the probability of occurrence for the given species. We ran ten replicates with cross-validation as the replicate run type, using a random starting seed for each replicate. We kept all other parameters at their default values. Finally, we used the mean from all replicates to analyze the final output.

## Supplementary Material

evag123_Supplementary_Data

## Data Availability

All code used in this manuscript, software with version numbers and parameters, is available at the GitHub repository: https://github.com/Tsylvester8/Cglo-popgen. For unlisted parameters in a given tool, we used default values. All sequences used in this study have been submitted to Sequence Read Archive (SRA) under BioProject PRJNA1043134.
