# Supplementary material for: Population Structure of the Jewel Scarab Chrysina gloriosa
Source: Genome Biol Evol. 2026 May 20;18(6):evag123. doi: 10.1093/gbe/evag123 (PMC13227606; doi:10.1093/gbe/evag123)
Supplement: evag123_Supplementary_Data [file evag123_supplementary_data.docx]

# Supplementary figures


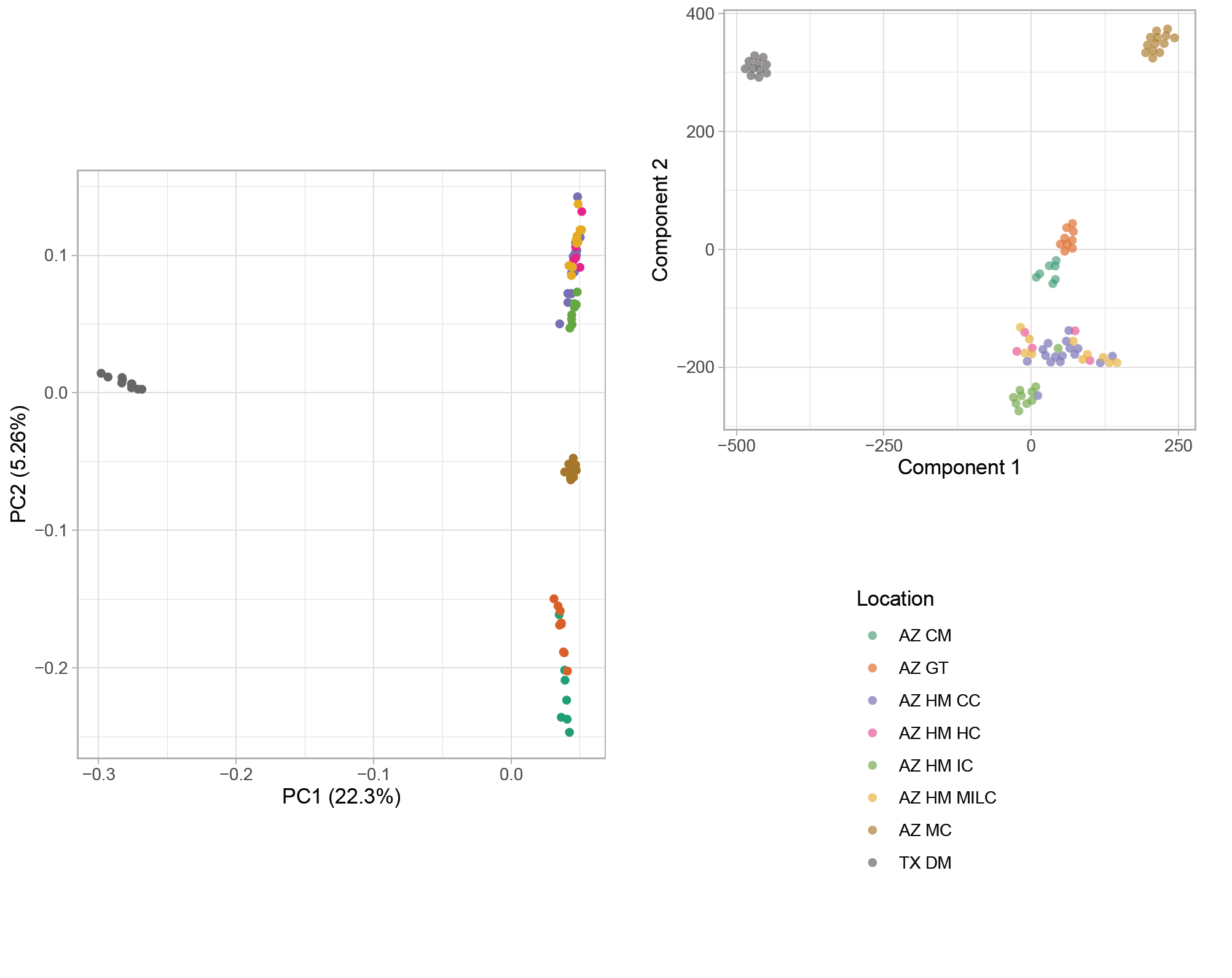


**Figure S1** **A)** Principal component analysis of nuclear only variants and **B)** t-SNE analysis of principal components. A clear separation between the Texas and Arizona specimens can be seen along the PC1 axis. Along the PC2 axis, we see clusters representing the three main regions of the mountain ranges. A fine-scale separation of populations can be seen in the t-SNE analysis.


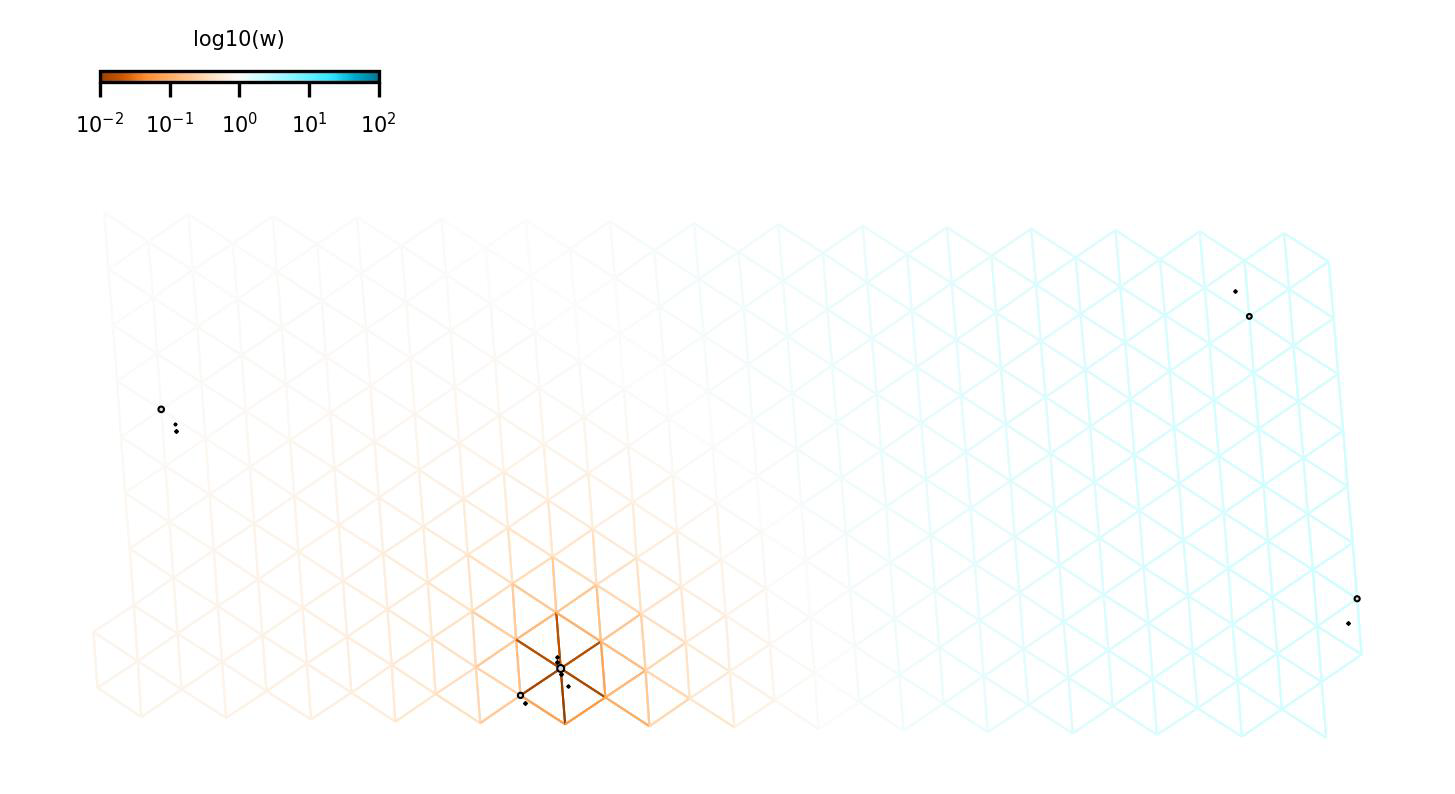


**Figure S2** Effective migration surface of *C. gloriosa* specimens from Arizona. Cooler colors (blue) represent increased probability of migration, while warmer colors (orange) represent decreased probability of migration.


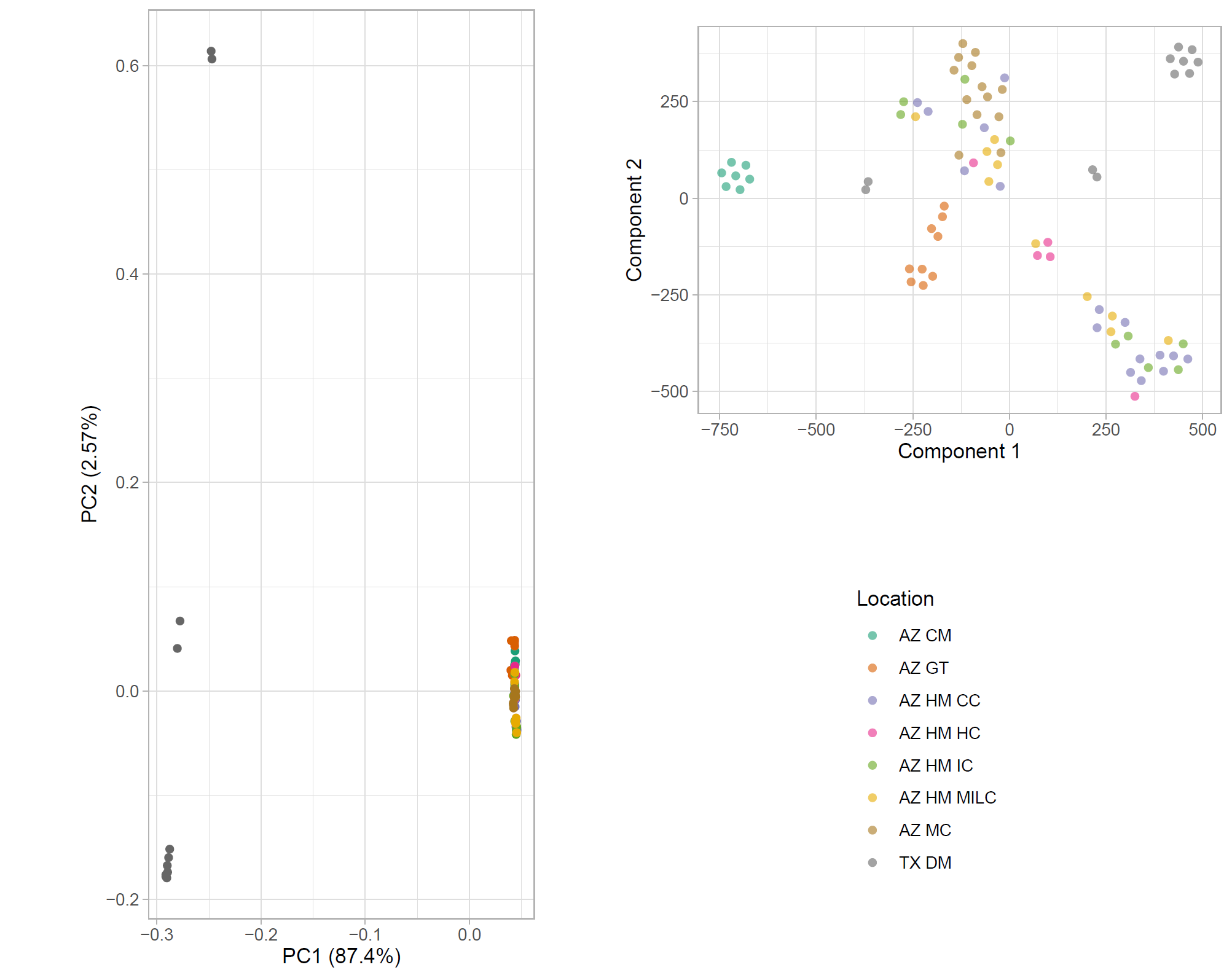


**Figure S3** **A)** Principal component analysis of mitochondrial only variants and **B)** t-SNE analysis of principal components. A clear separation between the Texas and Arizona specimens can be seen along the PC1 axis. Along the PC2 axis, there is less variation. However, when other principal components are taken into account, we can see a finer-scale separation of populations in the t-SNE analysis.


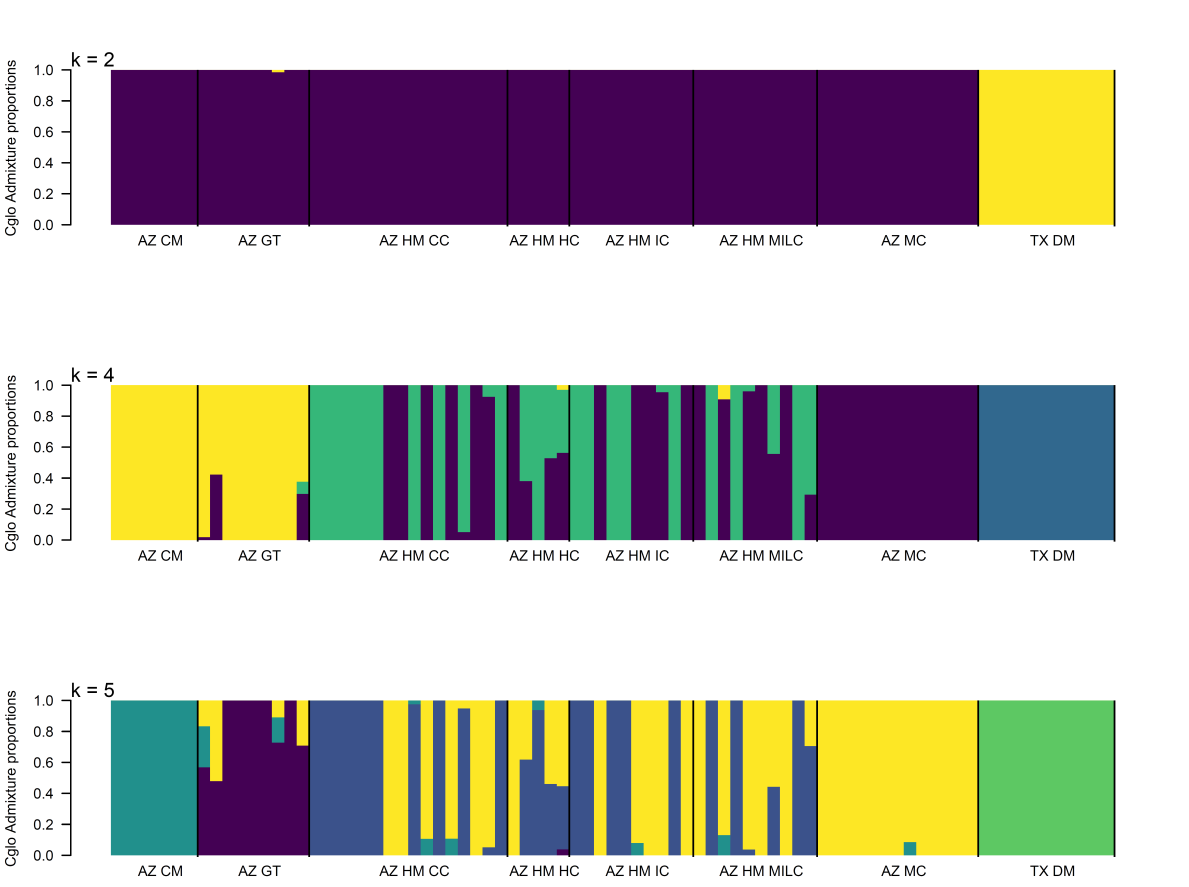


**Figure S4** Bayesian clustering of *C. gloriosa* populations with ADMIXTURE software using the mitochondrial variants. From top to bottom, bar plots of K = 2,4, and 5.


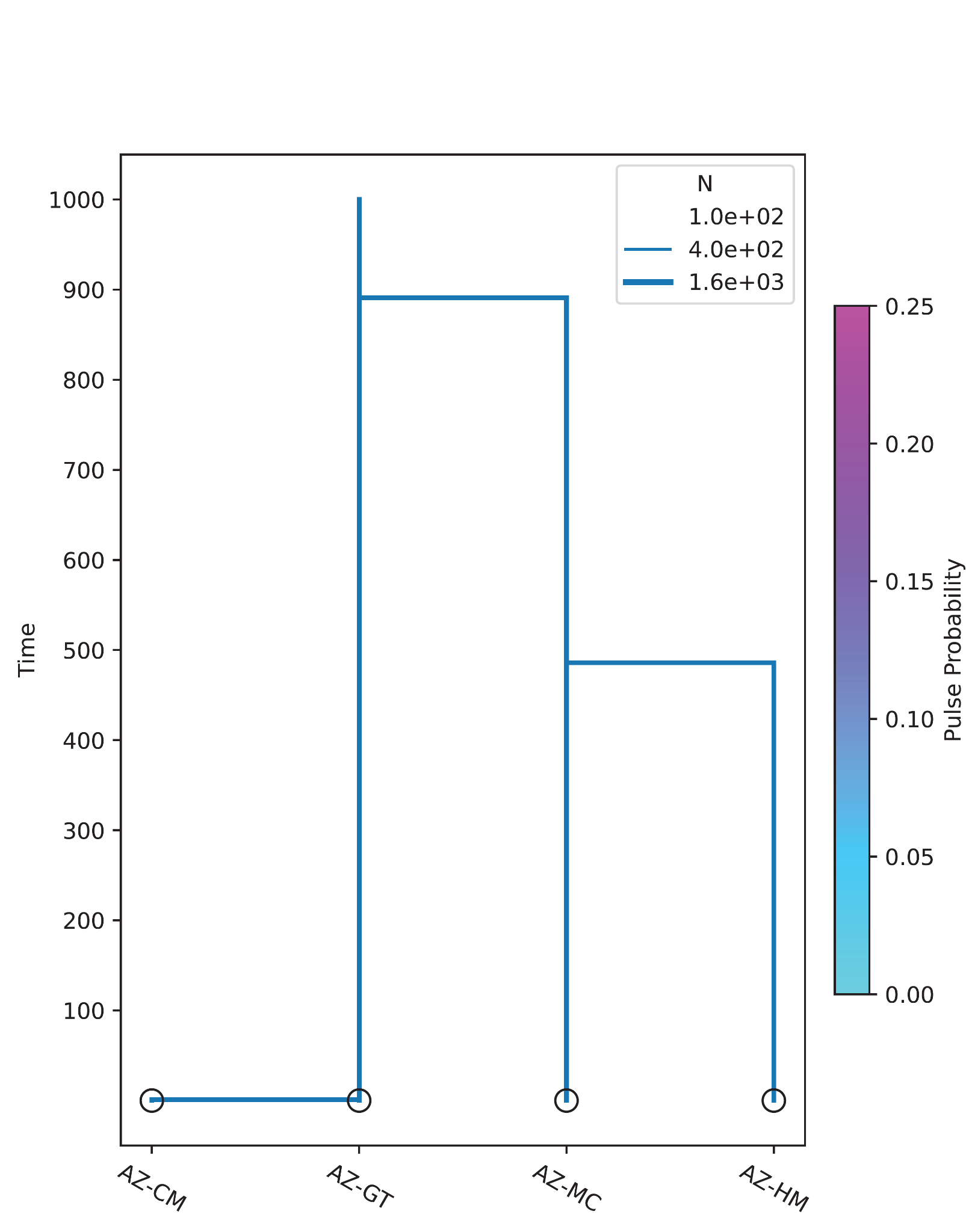


**Figure S5** Timing and pattern of population fragmentation in Arizona. Populations are labelled as follows: AZ-CM: Chiricahua Mountain; AZ-GT: Geronimo Trail; AZ-MC: Madera Canyon; AZ-HM: Huachuca Mountain. The Texas population separated from the Arizona population 56000 years ago (not included in this figure)


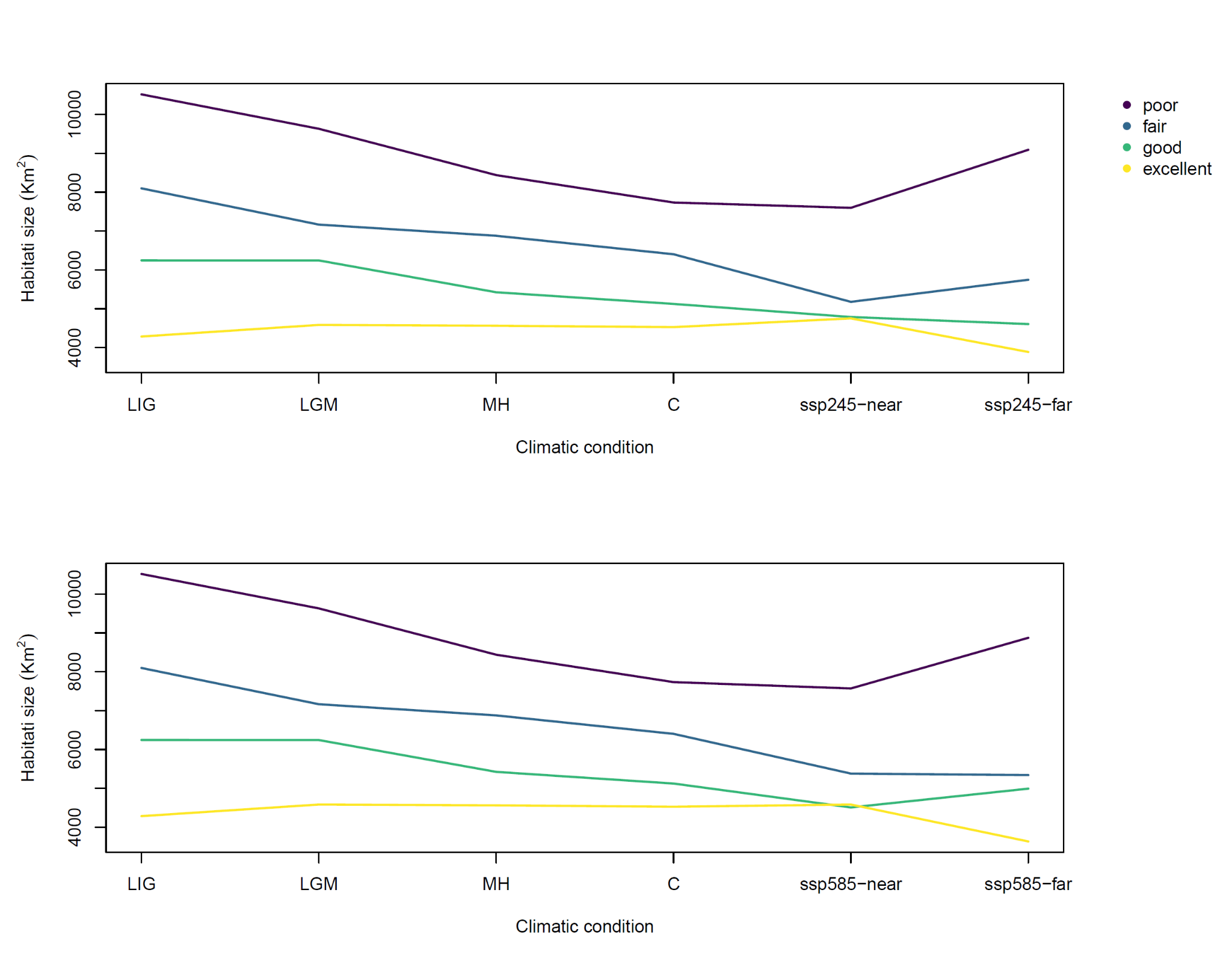


**Figure S6** Total suitable habitats of *C. gloriosa* at major climatic conditions. The habitat suitability is assessed based on the probability of observing the species and is binned as follows: poor (60% < and >= 70%), fair (70% < and >= 80%), good (80% < and >= 90%), and excellent ( > 90%). **Top** current and past climatic conditions and future climatic conditions predicted using the SSP245 emission pathway, and **bottom** current and past climatic conditions and future climatic conditions predicted using the SSP585 emission pathway.


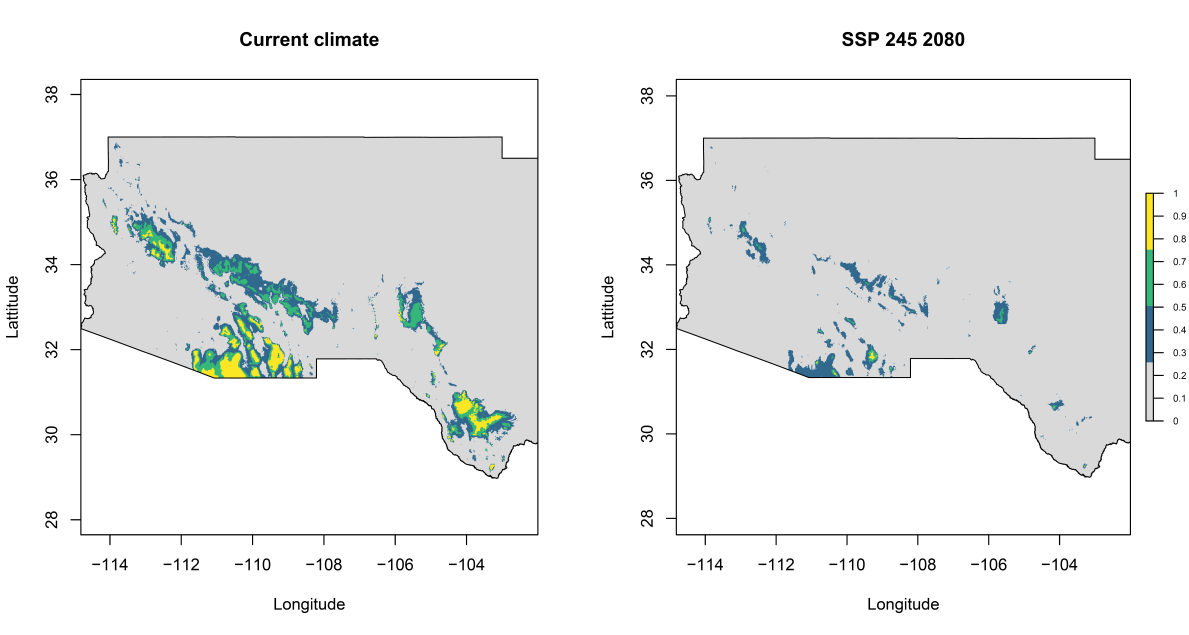


**Figure S7** Species distribution model of *C. gloriosa* under the present climatic conditions and future climatic conditions. We chose Shared Socioeconomic Pathway (SSP) 245 as the future climatic model.

# Supplementary Tables

**Table S1** Summary of population collection locations and approximate geographic coordinates

| **State** | **Mountain range** | **Sub population** | **Label** | **Count** | **Coordinates**  **(Latitude, Longitude)** |
| --- | --- | --- | --- | --- | --- |
| Arizona | Chiricahua Mountains |  | AZ-CM | 7 | 31.48998,  -109.0697 |
|  | Geronimo Trail |  | AZ-GT | 9 | 31.9117,  -109.2451 |
|  | Huachuca Mountains East | Carr Canyon | AZ-HM-CC | 16 | 31.4461,  -110.3105 |
|  |  | Miller Canyon | AZ-HM-MILC | 10 | 31.4391,  -110.282 |
|  |  | Hunter Canyon | AZ-HM-HC | 5 | 31.417,  -110.274 |
|  | Huachuca Mountains West | Ida Canyon | AZ-HM-IC | 10 | 31.38,  -110.33 |
|  | Madera Canyon |  | AZ-MC | 13 | 31.7262,  -110.875 |
| Texas | Davis Mountains |  | TX-DM | 11 | 30.706, -104.104 |

**Table S2** Summary of Pairwise Fst estimates

|  | **AZ-CM** | **AZ-GT** | **AZ-HM** | **AZ-MC** | **TX-DM** |
| --- | --- | --- | --- | --- | --- |
| **AZ-CM** | - |  |  |  |  |
| **AZ-GT** | 0.00586 | - |  |  |  |
| **AZ-HM** | 0.01077 | 0.0081938 | - |  |  |
| **AZ-MC** | 0.014905 | 0.012051 | 0.0077984 | - |  |
| **TX-DM** | 0.28376 | 0.28213 | 0.28609 | 0.2872 | - |

**Table S3** Average population heterozygosity (pi). The pi is averaged over 10,000-bp windows.

| **Population** | **pi** |
| --- | --- |
| AZ-CM | 0.000520908 |
| AZ-GT | 0.000478442 |
| AZ-HM | 0.000550648 |
| AZ-MC | 0.000507874 |
| TX-DM | 0.000630489 |
